# Mobile App Usage Patterns of Patients Prescribed a Smoking Cessation Medicine: Prospective Observational Study

**DOI:** 10.2196/mhealth.9115

**Published:** 2018-04-17

**Authors:** Marianna Bruno, Marcia Wright, Christine L Baker, Birol Emir, Eric Carda, Michelle Clausen, Catherine Sigler, Aanal Patel

**Affiliations:** ^1^ Pfizer New York, NY United States; ^2^ Pfizer Nashville, TN United States; ^3^ United BioSource Corporation Ann Arbor, MI United States; ^4^ Express Scripts Inc St Louis, MO United States

**Keywords:** smartphone, mobile apps, technology, patient engagement, patient satisfaction, patient adherence, surveys, smoking cessation

## Abstract

**Background:**

Cigarette smoking is the leading preventable cause of death and is responsible for more than 480,000 deaths per year in the United States. Smoking cessation is challenging for many patients. Regardless of available treatment options, most quit attempts are unaided, and it takes multiple attempts before a patient is successful. With the ever-increasing use of smartphones, mobile apps hold promise in supporting cessation efforts. This study evaluates the ease of use and user satisfaction with the Pfizer Meds app to support smoking cessation among patients prescribed varenicline (Chantix).

**Objective:**

Study participants included varenicline users who downloaded and used the app on their personal smartphone. The main objectives were to report mobile app download frequency and usage details and to describe the participant-reported satisfaction with and usefulness of the app over the 14-week follow-up study period.

**Methods:**

Adults aged 18 years or older who had been prescribed varenicline were identified from the Express Scripts Incorporated pharmacy claims database. After meeting privacy restrictions, subjects were sent an invitation letter and second reminder letter with instructions on how to download the Pfizer Meds mobile app. Participants received a push notification to complete a smartphone-enabled survey regarding the utility of the app 12 weeks after downloading the app. Descriptive statistics summarized sociodemographics, use of varenicline, and details of use and satisfaction with the mobile app.

**Results:**

Of the 38,129 varenicline users who were sent invitation letters, 1281 participants (3.35%) downloaded the Pfizer Meds app. Of the 1032 users with demographic and other data, 585 (56.68%) were females, and 446 (43.22%) were males; mean age was 46.4 years (SD 10.8). The mean number of app sessions per participant was 4.0 (SD 6.8). The end-of-study survey was completed by 131 survey respondents (10.23%, 131/1281); a large number of participants (117/131, 89.3%) reported being extremely, very, or moderately satisfied with the app. A total of 97 survey respondents (97/131, 74.0%) reported setting up a quit date in the app. Of those, 74 (74/97, 76%) reported quitting on their quit date.

**Conclusions:**

Positive patient engagement was observed in this study based on app download and usage. This study quantified how the Pfizer Meds app performed in an observational real-world data setting. The findings demonstrate the willingness of participants to set a quit date and use the app for support in medication adherence, refill reminders, and information regarding how to take the medication. This study provides real-world evidence of the contribution apps can make to the continued encouragement of smokers to improve their health by smoking cessation.

## Introduction

### Background

Cigarette smoking is the leading cause of premature death, causing approximately 480,000 deaths annually in the United States [1]. Although the negative health effects of smoking are well known, it remains a major preventable public health issue. It is estimated that approximately 16% of US adults aged 18 years and older currently smoke [2].

Although many smokers state a desire to quit, few are able to do so without help. Regardless of the available treatment options, more than half (52%) of quit attempts by US smokers are unaided attempts, with an average success rate as low as 5% [3]. A large number of studies have demonstrated the efficacy of medication and counseling to support smoking cessation [4].

### Smoking Cessation Apps

Mobile apps have been developed to assist with smoking cessation. Abroms et al systematically evaluated 47 iPhone apps for smoking cessation [5], characterizing them by app type (eg, calculator, rationing, or other), level of adherence to the US Public Health Service’s 2008 Clinical Practice Guideline for Treating Tobacco Use and Dependence [6], frequency of downloads, and app price. The authors found that as a group, the iPhone smoking cessation apps generally failed to follow the guidelines, to ask users for their tobacco use status, assess their willingness to quit, arrange for a follow-up, recommend the use of approved medications, and the use of counseling and medication to quit smoking.

Another group of investigators similarly reviewed the attributes of Facebook apps for smoking cessation [7]. A smaller group of apps (9) both met the search criteria and were available on Facebook. Similar to the findings from the iPhone app review, Facebook apps had a low level of adherence to the US Public Health Service’s 2008 Clinical Practice Guideline for Treating Tobacco Use and Dependence.

Using a different approach, Haskins et al [8] reviewed published research articles related to smoking cessation apps and looked in app stores for the apps that were evaluated in the literature. Using this approach, they were able to identify the available evidence-based apps, finding a total of 6 that were available in the app stores. The authors concluded that the process of finding evidence-based smoking cessation apps was quite difficult, and there is a need to continue to advance techniques to find technology-based health interventions.

To investigate the contents of smoking cessation apps in South Korea, Choi et al [9] searched Google Play and the Apple iTunes store for keywords “smoking” and “smoking cessation” in either Korean or English. Applying a priori criteria (eg, apps were selected if targeted to general consumers rather than physicians, not for hypnosis, not for simulation of smoking), they found a total of 309 apps to evaluate, and of these, they randomly selected 175 apps. Apps were then coded based on self-determination theory (focuses on stimulation of autonomous motivation). On the basis of their review, the authors concluded that smoking cessation apps generally might not sufficiently stimulate autonomous motivation (ie, autonomy, competence, and relatedness).

### Smoking Cessation Treatment

Pharmacotherapeutic approaches also play a key role in smoking cessation. This study was based on varenicline users, so it was important to consider the use of the app in patients prescribed varenicline. Varenicline is used as an aid to support smoking cessation, and its efficacy has been demonstrated in a number of randomized, controlled clinical studies, most recently in a large, prospective, randomized, double-blind, active- and placebo-controlled pharmacotherapy trial, which evaluated the neuropsychiatric safety and efficacy of varenicline, bupropion, and nicotine replacement therapy (NRT) patch versus placebo; varenicline showed superior efficacy to placebo (*P*<.001) and both bupropion and NRT patch (*P*<.001) at the end of treatment (weeks 9 through 12) and follow-up (weeks 9-24). Bupropion showed similar efficacy to NRT patch (*P*=.60), and both showed superior efficacy versus placebo (*P*<.001) [10]. Although pharmacological approaches have proven efficacy and are supported in the 2008 US Public Health Service (PHS) Guidelines on treating tobacco use and dependence, additional recommendations include combination therapy and social or behavioral support to increase cessation rates [6]. Therefore, it is prudent that clinicians understand and use multiple approaches, particularly to enhance and support the success of a smoking cessation attempt.

With the increasing convenience and wide reach of smartphones during the last decade [11], an increasing number of people are now taking advantage of smartphones to tackle health issues. Smartphones are mobile phones with advanced functionality and features. Mobile health (mHealth) apps have risen in popularity, providing new opportunities to change health-related behaviors and manage chronic conditions [12,13]. The World Health Organization defined mHealth as medical and public health practice supported by mobile devices, such as mobile phones, patient monitoring devices, personal digital assistants, and other wireless devices [14]. These health apps can provide immediate access to health information, medication reminders, as well as help track progress toward a health-related goal such as a weight loss regimen; however, many factors related to smartphone health and wellness app use are not yet fully understood.

Globally, smartphones are increasingly used in health information and health care delivery. As of 2016, the global number of mHealth apps had reached 259,000 apps. Today, there are more than 59,000 mHealth app publishers on the main app stores worldwide, and the trend is quickly rising [15]. In a wide range of countries, smoking cessation services are using mobile phones to help deliver support, particularly in conjunction with other services [16]. The potential benefits of mobile phone–based smoking cessation interventions include ease of use anywhere at any time; cost-effective delivery and scalability to large populations, regardless of location; and the ability to link the user with others for social support.

Concurrent with the rise in smartphone use to advance health goals such as smoking cessation, there has been increased emphasis on patient empowerment [17]. Patient educational programs have proliferated to provide health and wellness support through tools such as apps and including a focus on improving adherence to medications [18-23]. High levels of medication adherence are associated with better clinical outcomes, greater treatment satisfaction, better quality of life, and lower overall health care costs [24]. A recent publication by Laffer and Feldman indicates that some patients proactively seek out ways to improve their medication adherence, and this has included the use of reminder services and third-party apps on their mobile devices [25]. Such devices are becoming popular patient-driven routes of accessing information. However, limited information exists on patient access, use, and outcomes of product-specific mobile apps.

In support of smoking cessation, Pfizer developed a mobile app for use by varenicline users to provide educational information and support on quitting smoking with varenicline, including how to take the medication, possible side effects, and safety considerations in addition to motivational support. Before deciding to launch the app broadly in the United States, this study was conducted to better understand the utility of the app in the real-world setting. The objective of this pilot study was to characterize the participant population of varenicline users who opted to use the app and evaluate the app’s functional attributes by analyzing mobile app download frequency and usage as well as describe participant-reported satisfaction with and usefulness of the app over the approximate 14-week follow-up study period.

## Methods

### Study Design

This was a noninterventional, prospective study of individuals prescribed varenicline for use as an aid to smoking cessation treatment. Pharmacy claims data were gathered from the database of a pharmacy benefits manager Express Scripts Incorporated (ESI) to identify varenicline users to be sent an invitation letter for participation. Outreach was restricted to ESI client organizations who have agreed to the use of their members’ data for research purposes. Potential participants were provided a unique alphanumeric code in the invitation letter with instructions on how to download the mobile app from Google Play (app store for Android products) or iTunes (app store for iOS, iPhone Operating System, or Apple products). Once the app was downloaded by a potential study participant, the code was required to unlock app functionality.

Participants were excluded from the study if they were younger than age of majority at study enrollment (eg, aged <19 years in Alabama and Nebraska and aged <21 years in Puerto Rico) or if the participant did not agree to the Pfizer Meds Mobile App Informed Consent Document (ICD) and Privacy Notice (PN). After downloading the app and entering the unique code, informed consent for study participation was presented in the app screen window. Without agreement to ICD and PN, the participant was not able to progress further and unlock the full app functionality. Participants must also have agreed to the End User License Agreement (EULA) by scrolling to the bottom of the screen and checking the box next to the acceptance statement. Study participants received an invitation to complete a smartphone-enabled survey regarding the utility of the app after 12 weeks of use of the app.

### Data Sources and Measurement

There were four sources of study participant data, which included the following:

1. *Pfizer Meds mobile app downloads:* The number of downloads of the app was tracked by App Figures. App Figures is a product that provides download data for apps available on the Google Play and Apple iTunes stores.
2. *Pfizer Meds mobile app use:* The app captured metrics via Google Analytics related to the type of material the participant accessed and level and dates of activity within the app. Google Analytics provided mobile analytics reporting for all activities occurring within the Pfizer Meds app. All activities were associated with the user’s unique alphanumeric code and time stamped. Google Analytics used a number of components to ensure proper tracking of measurements of user interactions. Google Analytics tracked app installation, active users and demographics, screens and user engagements, and crashes and exceptions.
3. *ESI pharmacy claims:* Anonymized pharmacy claims data were analyzed via the ESI claims database and included age, gender, varenicline fill date(s), number of varenicline days’ supply per fill and refill, dose per fill and refill, and refill indicator. Varenicline claims data were collected and analyzed for the participant-specific study period.
4. *Participant smartphone-enabled survey responses:* The one-time survey was taken at a minimum of 12 weeks after enrollment and included questions related to participant sociodemographic characteristics (including ethnicity and race), varenicline knowledge, satisfaction with and usefulness of the app and information provided therein, smoking history, current smoking status, additional support services used to quit smoking, comorbid conditions, and intention to continue using the app after completion of the survey. Furthermore [26], the survey was reviewed and approved by the institutional review board. Before launching the survey, a thorough testing of its programming was conducted to ensure skip patterns were followed, question branching logic properly working, and data validations and error messages accurately programmed. As part of the survey development, test data were used to check data tabulations for validity. The survey programming was also tested using 2 methods to ensure accuracy: systematic and stochastic testing. Survey programmers followed a testing plan and tested the skip patterns and branching logic. Systematic testing was followed by a process of automated stochastic testing, which avoids assumptions of user bias, where testing software randomly chooses responses in an effort to test the scripting logic from end to end. Following the aforementioned testing, a live link to the survey was provided to testers who subsequently verified the content and presentation of the survey based on comparison with written plans.

Survey participants were a convenience sample, as individuals voluntarily took part in the follow-up survey. No personal information was collected or stored.

Data were reviewed following written data review guidelines that specified the visual data review to be performed on the listings and tabulations of participant survey data. Data were reviewed before database lock to exclude data if needed as a result of straight-line or overly rapid responses. Due to the cross-sectional nature and mode of administration of the survey, as well as protection of participant privacy, survey data were used as reported by participants (participants were not contacted to clarify responses).

### Patient Recruitment

Participant recruitment was done via the ESI pharmacy claims database; ESI is the largest pharmacy benefits manager in the United States, covering approximately 1 in 4 Americans. Their pharmacy claims database houses all pharmacy claims adjudicated for participants who have ESI as their pharmacy benefits manager. Only pharmacy claims for ESI clients that allow for deidentified data for use in mining activities, statistical compilation, or research were used in this study.

ESI identified all members, aged 18 years or older, who had received varenicline via mail order or retail pharmacy within the previous 30 days. This was the defined “lookback” period for participant recruitment. All such participants were issued a study invitation by email. These identified “index claims” were not required to be the first varenicline claim for the participant and may have been a refill prescription. This process was completed monthly for a period of 5 months, targeting different potential participants each month. Invitees received a second invitation (reminder) letter during the subsequent mailing.

### Study Period

The recruitment period started on the date of the first mailed study invitation letter and continued for 5 months. All eligible participants were invited to download the Pfizer Meds mobile app and to complete a cross-sectional smartphone-enabled survey no earlier than 12 weeks after the app download date. Moreover, 12 weeks correspond to the approved treatment length of varenicline [27].

The expected time from agreement with the ICD and PN to survey completion was 12-14 weeks for the last enrolled participant; those who enrolled earlier had a longer window for survey completion. The survey was closed after 14 weeks following enrollment of the last participant; as of that time, no further survey reminders were issued through the app.

### Study Size

The target sample size was set for up to 1000 enrolled participants. Up to 100,000 participants in the United States were planned to be invited to participate in this study to reach the target sample size. The projected response rate of 1% was considered a conservative estimate and was based on results of similar studies that used the same study recruitment method (mailed letter sent to nonblocked ESI members; personal communication LISA GRIBBLE, July 15, 2017). Recruitment rate was closely monitored, and number of mailed invitations was adjusted in case the target sample size of 1000 enrolled participants was reached before the end of recruitment.

### App Content and Functionality

The app included 20 unique pages, including set-up, a main screen, reminders, savings calculator, an information vault, badges, and notifications. Examples of some of the most frequently visited pages can be seen in Multimedia Appendix 1.

### Statistical Methods

Descriptive statistics, including mean, SD, minimum, first quartile, median, third quartile, and maximum values for continuous variables and numbers and percentages for categorical variables, were calculated to characterize study subjects who downloaded the app, including their use of and satisfaction with the mobile app.

The proportion of invited participants who downloaded the mobile app was calculated and summarized. Participants comprising the numerator were defined as those invitees who had unlocked the app with their unique ID and had made it through to the welcome screen, having accepted the terms of the ICD and PN and the EULA.

## Results

### Express Scripts Incorporated Varenicline Users, Invitation Letters, and Pfizer Meds App Downloads

**Figure 1:**
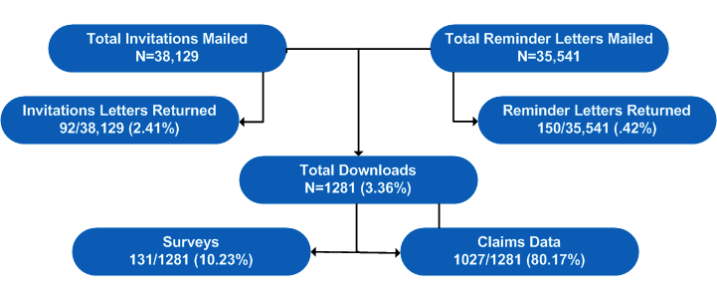
Study participation subgroups. Claims data were available for 1027 of the 1281 download participants’ data due to a change in Express Scripts Incorporated (ESI) policy during the study period.

A total of 38,129 initial invitation letters and 35,541 reminder letters were mailed to ESI varenicline users between September 2015 and February 2016 (see Figure 1). Of these, a total of 1281 participants downloaded the Pfizer Meds app (overall response rate of 3.36%). Of note, claims data could only be obtained from 1027 participants due to a change in the ESI policy during the study period.

Among the 1281 participants who downloaded the app, females represented a larger percentage (585/1281, 45.67%) compared with males (446/1281, 34.82%), and gender was missing for 19.52% (250/1281). For the 1032 users with known age, the mean age was 46.4 years (range 19-85 years), with the majority in the 35- to 65-year age group (671/1032, 65.02%).

### Pfizer Meds App Use Metrics

A key objective of this study was to evaluate features of the app. The Pfizer Meds app use metrics are provided in Table 1. The My Pfizer Meds page, the main screen that is displayed when the app is opened, was visited the most frequently, at an average of 4.1 times among the 1220 participants who ever visited that screen. From this page, the participant could swipe through product images and view content on how to take the medication, the full prescribing information, medication guide, and full safety information. The next most frequently visited pages (and the number of times visited on average) were My Info Vault (3.8), Unlock (3.3), Reminder Display (2.8), and Reminder Set (2.7).

The “my information vault” page, the central repository of patient educational information, was viewed by approximately half of the enrolled population (51.52%, 660/1281). Those participants who viewed “my information vault” primarily accessed slip-ups, tips for handling urges, and also celebrating quitting windows. Participants rarely viewed windows associated with medication-related items, such as how to take my medication, starting my medication, and the most important safety information I should know about my medication. This could be due in part to the recruitment methodology whereby patients were identified for potential participation based on a filled claim for varenicline. Therefore, it is expected that most participants had already decided to start medication or started taking their medication before downloading the mobile app. In addition, this also reflects the challenge of smoking cessation and support patients may need during their quit attempt with medication.

Participants generally displayed a positive level of engagement to various app features. A large proportion of participants set up a quit date (1086/1281, 84.77%) and refill reminder at 1 month (1134/1281, 88.52%). More than half of enrolled participants (717/1281, 55.967%) set up motivational notifications. In contrast, dosing reminder activity utilization within the app was relatively low (109/1281, 8.51%).

Badges were earned as milestones and were reached for prespecified activity in the app. Badges were most often earned for designating a quit date (925/1281, 72.21%) and setting a reminder to take meds (679/1281, 53.01%).

### Twelve-Week Postenrollment Survey Results

A total of 131 respondents (131/1281, 10.23%) completed the end-of-study survey. Table 2 provides data on survey respondents’ smoking history and smoking status. The mean number of years smoked by survey respondents was 22.1 (SD 9.9) years. The majority of respondents (55.0%) indicated that aside from the current attempt, they previously tried to quit smoking fewer than 6 times. In addition to varenicline and the Pfizer Meds app, participants reported using family and friends (58.8%) and health care providers (HCPs; 40/131, 30.5%) as additional quit smoking support resources; 22.9% (30/131) of respondents only used the Pfizer Meds app.

When asked about their satisfaction with the Pfizer Meds app, a large proportion of participants (89.3%, 117/131) reported being extremely, very, or moderately satisfied with the app. In addition, 54.2% (71/131) rated the usefulness of the app in terms of supporting their smoking cessation goal as extremely or very useful. Only one subject indicated that the app was not at all useful.

In response to questions regarding the assessment of the usefulness of various components of the Pfizer Meds app, more than half of the 131 survey respondents found reminders to take the medication, medication refill reminders, and information about how to take the medication (78/131, 59.6%; 70/131, 53.4%; and 76/131, 58.1%, respectively) to be extremely or very useful. A similar number of respondents found the savings calculator and the message notifications (77/131, 58.8%, and 76/131, 58.0%, respectively) to be extremely or very useful. When asked about potential future use, 60.3% (79/131) of respondents reported planning to continue using the app in the future.

A total of 97 survey respondents (97/131, 74.0%) reported setting up a quit date in the app. Of those, 74 (74/97, 76%) reported to have stopped smoking on the chosen quit date. Regarding intentions, 68.7% (90/131) of survey respondents who set up a quit date reported feeling extremely or very confident that this quit attempt will be successful.

**Table 1:** Frequency of use metrics of the Pfizer Meds app. iOS: iPhone Operating System; OS: operating system.

Variable		Participant utilization (N=1281)
**Type of mobile on OS, n (%)^a^**		
	Android	672 (52.46)
	iOS	612 (47.78)
**Number of sessions per participant**		
	n (%)	1281 (100.00)
	Mean (SD)	4.0 (6.8)
	Median (min, max)	1.0 (1, 89)
**Number of times my medication page viewed per participant**		
	n (%)	1220 (95.24)
	Mean (SD)	4.1 (5.9)
	Median (min, max)	2.0 (1, 87)
**Number of times reminder set/user settings up page viewed per participant**			
	n (%)	1259 (98.28)
	Mean (SD)	2.7 (1.9)
	Median (min, max)	2.0 (1, 17)
**Number of times reminder display page viewed per participant**			
	n (%)	672 (52.26)
	Mean (SD)	2.8 (4.2)
	Median (min, max)	1.0 (1, 68)
**Number of times savings calculator set up page viewed per participant**			
	n (%)	1210 (94.46)
	Mean (SD)	1.3 (0.9)
	Median (min, max)	1.0 (1, 9)
**Number of times savings calculator graph viewed per participant**			
	n (%)	1175 (91.73)
	Mean (SD)	1.4 (1.0)
	Median (min, max)	1.0 (1, 11)
**Number of times main my information vault page viewed per participant**			
	n (%)	660 (51.52)
	Mean (SD)	3.8 (4.1)
	Median (min, max)	2.0 (1, 40)
**Number of times product images viewed per participant**			
	n (%)	425 (33.18)
	Mean (SD)	1.3 (0.6)
	Median (min, max)	1.0 (1, 4)
**Number of times important safety information expanded per participant**			
	n (%)	262 (20.45)
	Mean (SD)	1.1 (0.4)
	Median (min, max)	1.0 (1, 4)
**Number of times badges page viewed per participant**			
	n (%)	498 (38.88)
	Mean (SD)	2.3 (3.0)
	Median (min, max)	1.0 (1, 30)

^a^Percentages can sum to more than 100% because a participant can report more than one type of mobile OS.

**Table 2:** Smoking history and smoking status among survey respondents.

Variable		Survey respodents (N=131)
**What is the approximate total number of years you have smoked in your lifetime?**			
	n (%)	131 (100%)
	Mean (SD)	22.1 (9.9)
	Median (min, max)	20.0 (2, 45)
**In your lifetime, how many times have you tried to quit smoking (not including this time)? n (%)**			
	0 times, this is my first attempt at quitting	3 (2.3)
	Fewer than 6 times	72 (55.0)
	6-10 times	41 (31.3)
	More than 10 times	15 (11.5)
**Which of the following methods have you used in the past to help you quit smoking? (Select all that apply), n (%)**			
	Prescription medication	70 (53.4)
	Nicotine patches	63 (48.1)
	Nicotine gum	49 (37.4)
	Nicotine lozenges	13 (9.9)
	Nicotine inhaler	4 (3.1)
	Nicotine nasal spray	1 (0.8)
	E-cigarette	54 (41.2)
	Counseling advice	17 (13.0)
	Helpline	9 (6.9)
	Self-help materials	13 (9.9)
	Others (eg, hypnosis, acupuncture)	16 (12.2)
	Nothing (I quit “cold turkey”)	30 (22.9)
**What types of support resources did you use during this quit attempt in addition to the Pfizer Meds Mobile App? (Select all that apply), n (%)**			
	Family and friends	77 (58.8)
	Support group	12 (9.2)
	Health care professional (doctor, nurse, pharmacist)	40 (30.5)
	Quitline	9 (6.9)
	Self-help materials	24 (18.3)
	Program through my employer	9 (6.9)
	Web-based resources	15 (11.5)
	Another smartphone app	11 (8.4)
	Hypnosis	3 (2.3)
	Acupuncture	1 (0.8)
	Others	5 (3.8)
	None	30 (22.9)

## Discussion

### Principal Findings

This pilot study combined several data sources to capture and quantify real-world usage patterns of a patient-focused app among subjects prescribed a smoking cessation medication. The study successfully enrolled 1281 participants, achieving an overall response higher than the expected (3.36% vs 1% per protocol based on similar studies conducted within the pharmacy benefits manager).

Most commonly, participants earned badges for designating a quit date and setting up a dosing reminder for medication, highlighting interest in this type of functionality of the app. Interaction of participants with the app was less with medication activity–related pages. For example, 467 participants (36.46%) accessed how to take varenicline medication. It is worth noting that, according to the varenicline prescribing information, before initiating medication, patients are instructed to set a quit date and are advised to initiate varenicline about 1 week before the quit date. These results are aligned with the support motivated smokers need to reach their smoking cessation goal. The lower level of interaction with medication-specific app content might suggest that patients feel a level of comfort and reliability of using the set-up reminders to adjust and take the medication. This may also be a reflection of positive patient counseling by HCPs on prescribing the medication as well as the dispensing package of the product with instructions on how to take the medication.

Counseling and support is a critical component of smoking cessation. The 2008 US PHS guidelines [6] encourage behavioral support and patient engagement. The guidelines also state that “There is a need for innovative and more effective counseling strategies.” The results of this study indicate that the use of a branded mobile app for smoking cessation may support engagement for motivated smokers.

This study was unique in that it was a proof of concept pilot for the Branded Pfizer Meds mobile app, providing data on its use and participant-reported satisfaction in a real-world setting. Although patients were on a prescription product, the intervention was the delivery of educational material and support with the mobile app. The app was available for both iOS and Android platforms and has subsequently been removed.

A diverse array of data sources was used in this analysis. First, this study used the pharmacy claims data from ESI, the largest pharmacy benefit manager in the United States, allowing the study team to reach a diverse sample of potential participants. The study also collected a large volume of quantitative and qualitative information by combining data from various sources (Google Analytics, anonymized Express Scripts pharmacy claims data, and participant smartphone-enabled survey responses **)** to fulfill the study objectives.

Advantages associated with the smartphone-enabled survey that was accessible via the Pfizer Meds app included increased accuracy of data entry relative to paper surveys, timely collection of participant responses, and a relative cost reduction compared with survey administration by a separate stand-alone data source.

### Limitations

There are a few limitations to be mentioned. This study was not fully representative of smokers who wish to stop smoking, in large part due to the protocol’s inclusion criteria. In addition to being an adult, to be included in this study, a participant must have had access to a smartphone, been in the ESI sampling frame that included claims (primarily employer-based plans), and be able to download a mobile app. The percentage of participants who were mailed an invitation letter and who had access to and use smartphone technology is unknown.

In addition, the study period varied per participant, with those who enrolled earlier having a longer window for survey completion, which in turn may increase the probability of recall bias. In addition, participants who disabled their screen notifications either on their mobile device or within the app did not receive the notifications regarding survey completion. The usefulness of the participant survey data in relation to their experience with the mobile app may be limited for participants who were less active within the mobile app.

Another limitation of this study was that the Pfizer Meds app was only available in the English language and was only available to Android and Apple device users. Although these operating systems (OSs) account for 90% of the market share [15], this limited availability of the app excluded the participation of those using mobile devices under the umbrella of Windows and Blackberry (OS).

The smaller (n=131) group of participants who took the end-of-study survey were self-selected, and the majority of survey participants were of white ethnicity (88.5%). This may be a limit to generalizability, although these findings are in accordance with a cohort study that looked at predictors of cessation intervention websites use showing that non-Hispanic whites are more likely to visit and participate in Web-based smoking cessation programs [28]. Future studies should consider including attempts to reach varenicline users who were contacted but did not download the app to evaluate differences between participants and non-participants that may have impacted smoking cessation behaviors.

### Conclusions

Smoking is the leading preventable cause of death in the United States [29]. Various approaches to smoking cessation are available to assist smokers who wish to quit. A systematic review of literature regarding the effectiveness of approaches to smoking cessation was the basis of the US Preventive Services Task Force recommendations that include such interventions as pharmacological intervention, brief counseling by HCPs, and use of mobile apps for smoking cessation support [30].

This study is innovative in seeking to gain insight into usage patterns in a smoking cessation app designed to be used in conjunction with varenicline. The 3.4% response rate of downloading the app is encouraging, given the potential advantage of supplementing smoking cessation medication with more personalized patient education through this app.

The findings demonstrate the willingness of participants to set a quit date and use the app for support in medication adherence, refill reminders, and information regarding how to take the medication. A subset of smokers who wish to quit will benefit from the availability and continual refinement of educational apps. Using metrics available from smartphone use and a patient survey at 12 weeks after enrollment, this study quantified how this app performed in a real-world data setting and may guide further refinement to the app. Specifically, modifications may include improved app functionality and features that this work has found to be important to users with health information needs.

## Appendix

Multimedia Appendix 1 Screenshots of Pfizer Meds App.

## Abbreviations

ESI: Express Scripts Incorporated
EULA: End-User Licensing Agreement
HCPs: health care providers
ICD: informed consent document
iOS: iPhone operating system
mHealth: mobile health
OS: operating system
PHS: public health service
PN: privacy notice
