# Supplementary material for: Mobile App Usage Patterns of Patients Prescribed a Smoking Cessation Medicine: Prospective Observational Study
Source: JMIR Mhealth Uhealth. 2018 Apr 17;6(4):e97. doi: 10.2196/mhealth.9115 (PMC5930175; doi:10.2196/mhealth.9115)
Supplement: Multimedia Appendix 1 [file mhealth_v6i4e97_app1.pdf]

Multimedia Appendix 1 –frequently visited Pfizermeds App Screenshots

**Type of Material Accessed**	**Description of Material**	**Content of Page**
Welcome Screen to Enter Unique Unlock Code	The welcome screen allowed the participant to enter a unique unlock code from participant letter. Only valid unlock codes allowed the participant to proceed to accept requirements for study participation and the app functionality.	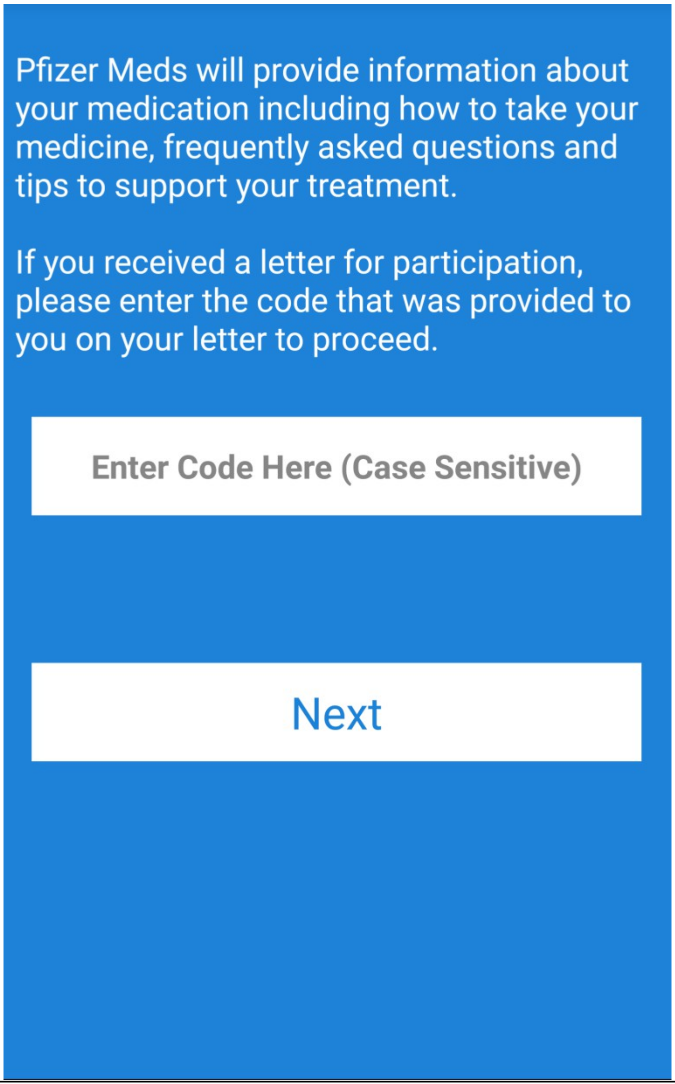

Multimedia Appendix 1 –frequently visited Pfizermeds App Screenshots

Type of Material Accessed	Description of Material	Content of Page
App Settings	The following settings could be updated to customize and set preferences for the app. The settings screen could be accessed after initial setup for the participant to change values at a later time.<br>• Remind Me – Set reminders for dosage frequency.<br>• Quit Date – The day the participant planned to stop smoking completely.<br>• Refill Reminder – Reminded the participant when to refill.<br>• Motivational Messages – Display motivational notifications during the participant's treatment.<br>• Tips to Handle Smoking Urges – Displayed weekly notifications to help overcome	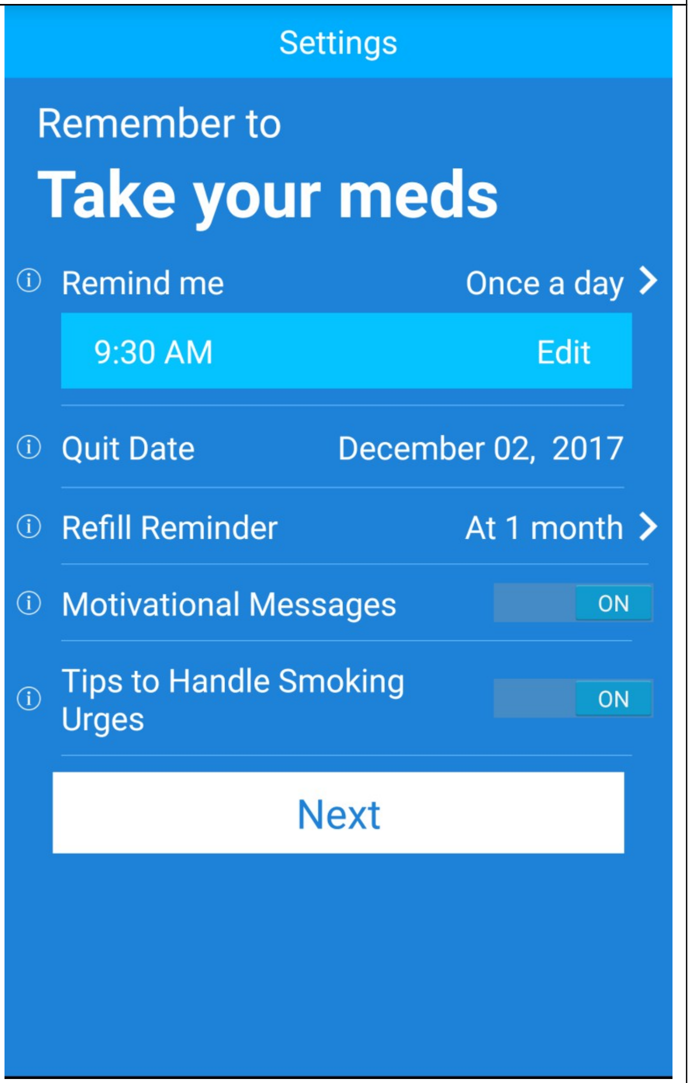

**Type of Material Accessed**	**Description of Material**	**Content of Page**
	urges.	
Reminders Display	If reminders were set, this screen was displayed under Reminders. The participant could scroll through past and future reminders and see when medication was taken. Medications marked as taken through reminders were automatically checked. The participant could also manually check or uncheck the item to indicate medication taken/not taken.	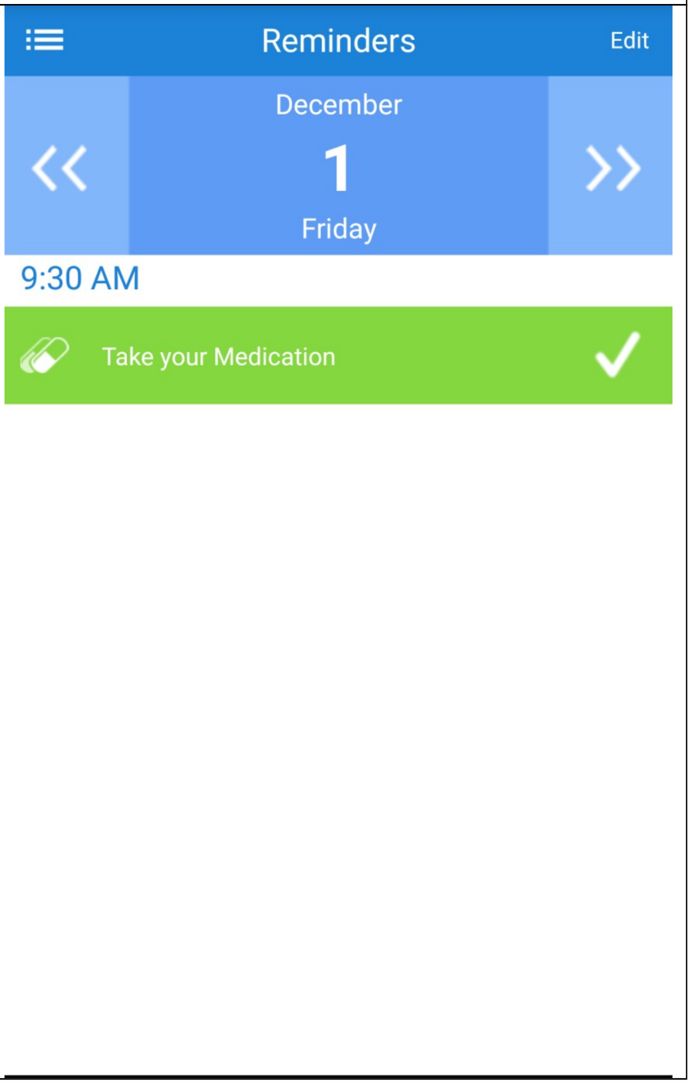

Multimedia Appendix 1 –frequently visited Pfizermeds App Screenshots

Type of Material Accessed	Description of Material	Content of Page
My Information Vault	List of resource categories were displayed under My Information Vault. The participant could view helpful resources by selecting a category or conducting a search.	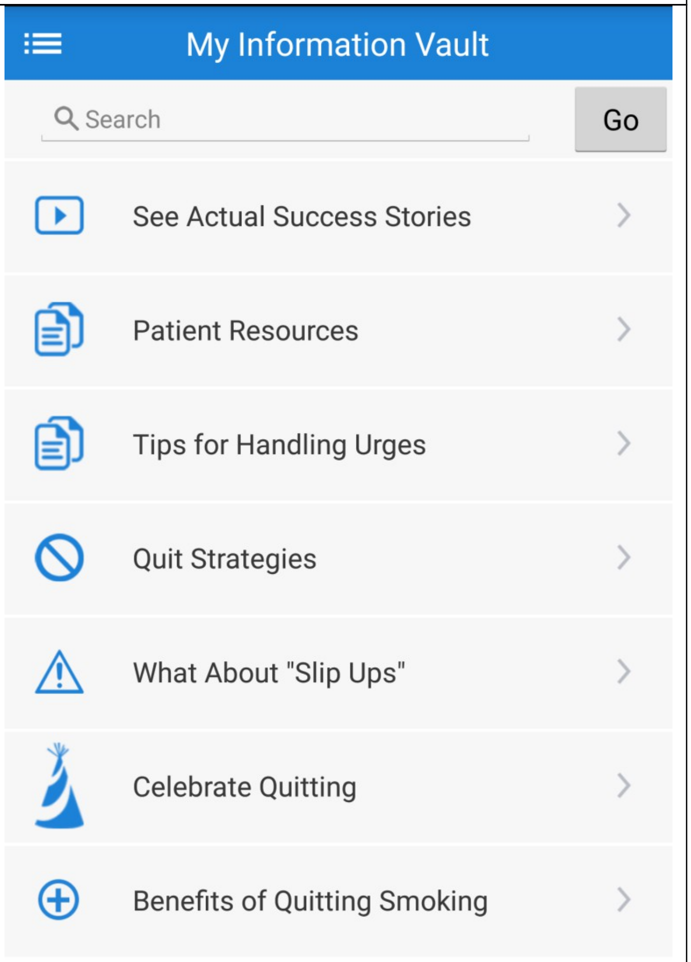

Multimedia Appendix 1 –frequently visited Pfizermeds App Screenshots

Type of Material Accessed	Description of Material	Content of Page
Badge Awarded	A badge was displayed when specific milestones were completed by the user.	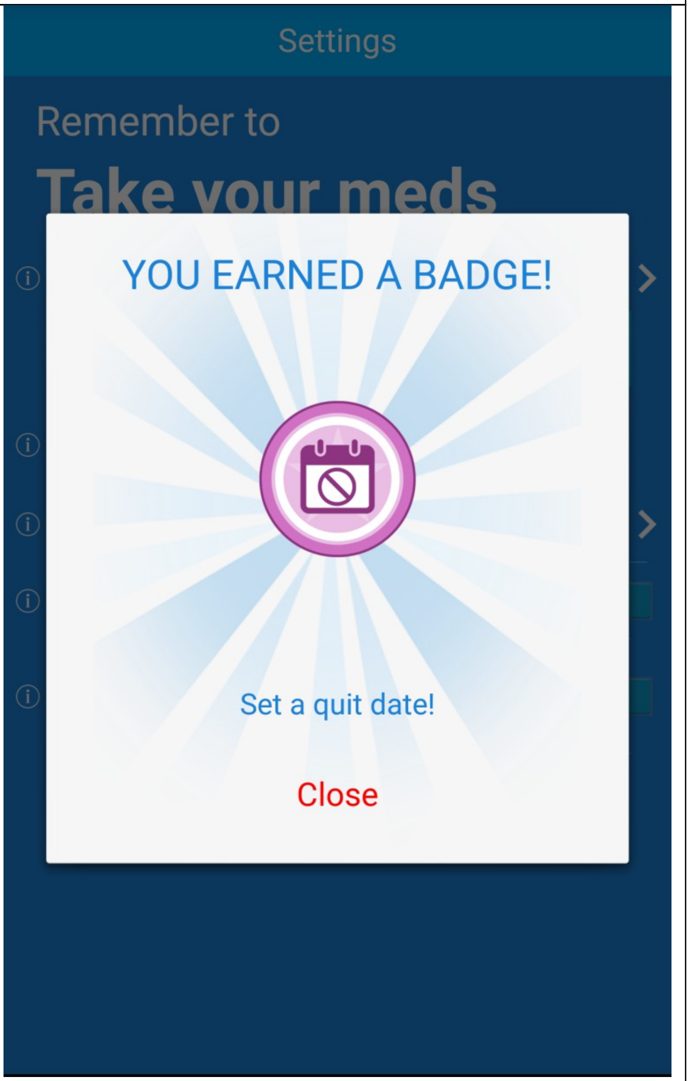
